# Validation of a measure of health-related production loss: construct validity and responsiveness - a cohort study

**DOI:** 10.1186/s12889-015-2449-z

**Published:** 2015-11-19

**Authors:** Malin Lohela Karlsson, Hillevi Busch, Emmanuel Aboagye, Irene Jensen

**Affiliations:** Unit of Intervention and Implementation Research (IIR), Institute of Environmental Medicine (IMM), Karolinska Institutet, SE 171 77 Stockholm, Sweden

**Keywords:** Production loss, Health conditions, Work ability, Convergent validity, Responsiveness

## Abstract

**Background:**

The aim of this study is to evaluate the construct validity and responsiveness of a Swedish measure of health-related production loss as well as to investigate if there is a difference in the level of production loss within a population suffering from persistent back/neck pain and CMDs.

**Methods:**

The sample was drawn from a study that assessed employees’ health and working capacity in 74 health care units before and after intervention. The study included 692 patients who reported working the previous six months at baseline measurement, and who were also asked to answer questions related to health-related production loss. Health-related measures were general health derived from Short Form-12, health-related quality of life derived from EQ-5D, and work ability derived from the Work Ability Index (WAI). Convergent validity and external responsiveness were assessed using Spearman’s Rank Correlation Coefficient and a linear regression model, respectively.

**Results:**

The different measures of health showed a moderate-to-strong correlation with the measure of health-related production loss and fulfilled the criteria for construct validity. Changes in health and work ability led to significant changes in health-related production loss, which demonstrates external responsiveness. This result is valid for both the total population and for the two different subgroups that were evaluated.

**Conclusions:**

The present study shows that this measure of health-related production loss is a valid measure for capturing production loss due to illness, and that work ability is more strongly correlated with health-related production loss than people’s general health is. The result shows an average of about 50 % reduced production due to illness, with back pain being the most costly.

## Background

Reduced productivity at work due to ill health or work environment problems in the form of absence from work, as well as reduced performance while at work, could lead to production losses for both society and for companies. Losses related to ill health, referred to as health-related production loss hereafter, have been highlighted recently in research as well as in society. Sickness absence was previously the only factor considered when estimating the cost of production loss from both the societal and employer perspective. However, more recent studies have shown that the costs related to consequences of employees attending work despite being sick, so-called presenteeism, could be at least twice as high as the cost of sickness absence [1, 2]. Thus the cost of production loss due to presenteeism has to be considered when production losses are estimated even from the broader societal perspective.

The practical concern, however, has been how lost production due to ill health is measured [3, 4]. Objective methods, often adopting computer-based measurements of health-related production losses, have been used sparingly due to the lack of generalizability of outcomes and to time constraints on implementation [5]. Instead, the use of self-reported productivity instruments is the most common approach [4, 5]. These self-reported instruments mostly measure indirect costs to the employer arising from health-related absences and reduced performance while sick but at work. To this end, efforts to measure productivity have focused on performance-based approaches to estimate health-related production loss in order to establish the business value of an employee’s health as well as to communicate to employers the reasons for investing in employee health [6–8].

Several productivity valuation instruments have been developed to capture these losses, some validated and others not [5, 8, 9]. The challenges, though, lie in the feasibility of collecting information related to production loss and how these instruments perform with regard to measuring productivity-related information, their applicability to real-world businesses, and the possibility of monetizing health-related production loss [5]. The measurement of production loss as a result of presenteeism seems to pose more challenges than the measurement of production loss due to absenteeism because production is not easily measurable in most cases [8, 10]. Furthermore, some of the instruments have been developed for a specific purpose directed towards particular groups and with varying recall periods also affecting the possibility to capture these losses [5, 8].

The implication of these challenges, from the employer’s perspective, is that measuring production loss is mired in uncertainty as to which measure will accurately capture the losses. To be useful, a measuring instrument must therefore: 1) determine if and to what extent health and work environment problems affect employee performance; 2) be possible to use when evaluating change over time as part of an intervention evaluation [3]; and 3) be capable of measuring the costs arising from the particular problem. Such a tool, of course, needs to be tested for its validity and reliability. A first step is to make sure that the instruments used capture what they are intended to capture, which is evaluated in different validation tests.

Health-related production loss, as measured in this study, has previously been tested for its construct validity in a working population [11]. That study indicated that health-related production loss was explained to a larger extent by health than by work environment-related factors. However, this difference was not statistically significant and the construct validity of this measure could therefore not be stated [11]. A possible explanation for this result is that health-related production loss also is a consequence of work environment-related problems, as shown in previous studies [12, 13], and it is questionable if the hypothesis that health-related production loss can be explained by health alone, without influence from work environment factors, was judiciously chosen. The current study expands the validation process for this Swedish measure developed to capture health-related production loss by examining its construct validity, and also by examining responsiveness validity in a population suffering from persistent back/neck pain or common mental disorders (CMDs). To date, these diagnoses are the two most common causes of sickness absence in Sweden as well as in Europe generally, and there has been a lot of focus on these groups for the prevention of sickness absence or the rehabilitation of those already on sick leave. These groups were also the targets of a large, nationally funded rehabilitation project launched in Sweden in 2009, from which data for this study has been drawn.

The specific aim of this study is to evaluate the construct validity and responsiveness of this measure of health-related production loss. A further aim of the study is to investigate if there is a difference in the level of production loss within a population suffering from persistent back/neck pain and CMDs.

### Convergent validity

Construct validity refers to the extent to which a construct measures the construct it is supposed to measure [14]. In this study, construct validity was tested by evaluating expectations about the relationship between different health-related conditions and health-related production loss (convergent validity). The validation of a measurement is a process that includes several steps depending on the characteristics of the measurement and what it is intended to be used for. The measurement evaluated here refers to work performance, or production loss, as well as to those factors that may limit this performance. In this part of the validation process, the focus is on the later part, i.e., whether the measurements are able to reflect or be indicative of health-related factors that may limit work performance. Work performance per se was not included in this data collection and is therefore not included in this validation. However, work ability was measured and is used as an indicator of performance, as it is related to employees’ ability to perform at work.

The measure of health-related production loss was correlated with different measures of health: general health, health-related quality of life, and work ability. If these three constructs correlate with health-related production loss as defined in the hypothesis below, this indicates convergent validity. First, health-related production loss was correlated with a question about general health from the validated questionnaire Short Form-12 (SF-12) [15]. Previous studies have shown that general health is weakly-to-strongly correlated with health-related production loss [9, 16]. This difference in the strength of correlation was found for different time points within the same study population [16], but was also related to the different instruments capturing health-related production loss [9].

#### Hypothesis H1a

General health is expected to have a moderate negative correlation with health-related production loss (r = −0.30 – -0.50).

#### Hypothesis H1b

General health is expected to have a moderate negative correlation with health-related production loss in patients suffering from N/B pain (r = −0.30 – -0.50).

#### Hypothesis H1c

General health is expected to have a moderate negative correlation with health-related production loss in patients suffering from CMD (r = −0.30 – -0.50).

Second, production loss was correlated with health-related quality of life (HRQL), measured by the validated Euroqol questionnaire EQ-5D [17]. HRQL has previously been shown to be associated with productivity [18, 19]. The degree of correlation has not been expressed. However, as HRQL is a measure that can be used to capture general health we assumed that the strength of the correlation for HRQL would equal that of general health.

#### Hypothesis H2a

HRQL is expected to have a moderate negative correlation with health-related production loss (r = −0.30 – -0.50).

#### Hypothesis H2b

HRQL is expected to have a moderate negative correlation with health-related production loss in patients suffering from N/B pain (r = −0.30 – -0.50).

#### Hypothesis H2c

HRQL is expected to have a moderate negative correlation with health-related production loss in patients suffering from CMD (r = −0.30 – -0.50).

Third, health-related production loss was correlated with the person’s overall work ability, measured using a question from the Work Ability Index (WAI) [20, 21]. Work ability is normally used as an indicator of an employee’s ability to perform at work but has seldom been evaluated in relation to a measurement of health-related production loss with data collected from the same population. One study has tried to evaluate if work ability is a robust indicator for assessing production loss [22]. However, this study used estimates of work ability from one population and modeled it with data on production loss from another population. The result from the study suggested that work ability is a robust indicator for assessing production loss. Two other studies [23, 24] evaluated the validity of a different measurement of how people function at work when experiencing health-related problems, The Work Role Functioning Questionnaire (WRFQ), and correlated it with WAI. They found a moderate to strong correlation between the two constructs. Based on the results in these studies we assume that work ability is at least moderately correlated with health-related production loss.

#### Hypothesis H3a

Work ability is expected to have at least a moderate negative correlation with health-related production loss (r = −0.30 – -0.50).

#### Hypothesis H3b

Work ability is expected to have at least a moderate negative correlation with health-related production loss in patients suffering from N/B pain (r = −0.30 – -0.50).

#### Hypothesis H3c

Work ability is expected to have at least a moderate negative correlation with health-related production loss in patients suffering from CMD (r = −0.30 – -0.50).

### Responsiveness

Responsiveness refers to the ability of an instrument to detect important changes either in terms of its ability to change over time, or in terms of how changes to it relate to corresponding changes in a reference measurement [25]. The first type of responsiveness is referred to as “internal responsiveness,” whereas the second type of responsiveness is referred to as “external responsiveness.” In this study, we evaluated the responsiveness of the measure using external responsiveness, i.e., whether changes in the scores of health-related production loss should be related to corresponding changes in various measures of health [25]. For all hypotheses (H4a-c) it was expected that health-related production loss would be reduced when health was improved, i.e., the different constructs were expected to have a negative association with health-related production loss.

#### Hypothesis H4a

Changes in general health are significantly and negatively associated with changes in health-related production loss.

#### Hypothesis H4b

Changes in HRQL are significantly and negatively associated with changes in health-related production loss.

#### Hypothesis H4c

Changes in work ability are significantly and negatively associated with changes in health-related production loss.

## Methods

In 2009, a national implementation of the so-called “rehabilitation warranty” (RW) began in Sweden. The political aim of the RW was to reduce, and prevent, sickness absenteeism among people suffering from persistent back/neck pain or CMDs. To accomplish this goal, the Swedish government offered economic compensation to the county councils in order to stimulate the use of evidence-based rehabilitation, mainly in primary care. Multimodal rehabilitation was offered for pain syndromes and was given in teams of at least three professionals (typically a medical doctor, a physiotherapist and a psychologist). It was usually carried out 2–3 times a week over a period of 6–8 weeks. Patients suffering from CMD were given cognitive behavioral therapy, often 8–10 sessions. Therapists offering cognitive behavioral therapy had to have at least a basic psychotherapy education, or be a licensed psychologist or psychotherapist. The RW encompasses all individuals of working age (16–67 years old) who are at risk of long-term sickness absenteeism due to persistent non-specific musculoskeletal pain or common mental disorders. The reason for focusing on these two diagnosis groups was that they represent the two most common causes for sick leave in Sweden. Since one of the goals concerns prevention, people not currently on sick leave may also take part in the interventions.

### Study design & population

A register-based study of the effects of the RW on sickness absenteeism was carried out in 2010 and included all individuals (*N* = 62,691) who had initiated treatment under the RW during the years 2009–2010. A sample of this population (*N* = 969) also participated in a survey, and received questionnaires to assess their health and working capacity before and after the interventions. Twelve county councils volunteered to participate in the survey. Seventy-four health care units (HCUs) offering interventions under the RW were selected, using a stratified random sampling procedure, based on type of HCU (public/private and primary care/specialized care). Patients were successively recruited by the staff at the selected HCUs. The staff was informed that all patients beginning interventions under the RW should receive written and oral information about the study and be asked to participate. Participation involved answering a brief questionnaire on HRQL and working capacity. The questionnaire was filled out twice: at the start of the rehabilitation and six months thereafter. The first questionnaire was distributed by the staff at the HCU, who also collected the patients’ informed consent. The second questionnaire was administered by the research team. Because the exact date of rehabilitation termination was unknown to the researchers, the follow-up questionnaire was sent out six months after the start of rehabilitation.

This study includes all patients who responded at the baseline measurement that they had had a job during the previous six months, since having a job was a prerequisite for patients’ being able to answer the questions.

### Ethical approval

The study was approved by the Central Ethical Review Board at Karolinska Institutet in Stockholm (Dnr 2009/1750-31/1).

## Measurements

Health-related production loss was measured with the question: *During the past seven days, how much did your health problems affect your performance while you were working? Think about days you were limited in the amount or kind of work you could do, days you accomplished less than you would have liked, or days you could not do your work as carefully as usual. If health problems affected your work only a little, choose a low number. Choose a high number if health problems affected your work a great deal.* The respondents were asked to rate how much their health-related problems had affected their performance using a scale from 0 to 10, where 0 = Health problems had no effect on my work, and 10 = Health problems completely prevented me from working. The question is based on one of the items in the Work Productivity Activity Impairment questionnaire (WPAI-GH) [16].

### Health conditions

General health was measured using a question from Short Form-12 (SF-12) [15, 26] (“In general, how would you say your health is”). The response options were ranged along a five-point scale from 1 (“excellent”) to 5 (“poor”). In the analyses, the scale was recoded into 1 = poor to 5 = excellent.

HRQL was measured using the EQ-5D [17]. The EQ-5D consists of five questions, answered on a three-point scale and covering the domains of physical mobility, hygiene, daily activity, pain, anxiety and depression. Answers can be transformed into a summary index ranging from 0 to 1, in which 0 signifies the worst possible health and 1 perfect health. Negative values could arise and needed to be dealt with in the analyses [27]. The Danish tariff was used to calculate the time trade-off scores (TTO scores) [28]. This index is a widely used measure of quality of life in different patient populations, allowing patient data to be compared with data from the general population.

Work ability was measured using a single item derived from the Work Ability Index (WAI) [20, 21], asking people to rate their present work ability compared to their lifetime best, on a scale from 0 to 10 where 0 represents totally incapacitated and 10 represents work ability when perceived as best. This single question has previously been shown to be highly correlated with the entire instrument [29].

## Statistical analyses

### Convergent validity

To test for convergent validity (the correlation between health-related production loss and different health-related measures), Spearman’s Rank Correlation Coefficient was used. This was chosen due to the scale of the variables in the test, where one variable is ordinal numeric and the other one is continuous. Correlations smaller than 0.1 were interpreted as no correlation, between 0.1 and 0.3 as a weak correlation, 0.3 to 0.5 as moderate, and 0.5 and above as a strong correlation [14]. The same categories were used for negative correlations. This analysis was performed using both the baseline and the follow-up measurement separately for both groups of patients, i.e., both CMD and back/neck pain patients. When analyzing HRQL, all individuals who received a negative score—i.e., perceived their health to be lower than 0—were excluded.

### Responsiveness

The responsiveness was assessed in terms of external responsiveness, that is, comparing changes in health-related production loss with changes in external standards [25]. Three measures of health were used as external standards in this study: HRQL, general health, and work ability. This part of the analyses included all patients who had had a job during the previous six months and had responded to both the baseline and follow-up measurement. All patients who received a HRQL lower than 0 (negative value) in any of the measurements were excluded from the analyses when evaluating the significance of changes in HRQL and health-related production loss. The analyses were performed using a linear regression model. Age, gender, educational level, and the subgroup (back/neck pain and CMD) were included in the regression models as covariates.

## Results

The total study population consisted of 965 patients. The participants had a mean age of 41 years (SD 12) and the majority were women (78 percent), see Table 1. Of these, 692 (72 %) reported at baseline that they had had a job during the previous six months and were therefore included in this study. 88 percent of the population suffering from CMD reported that their disorder resulted in production loss. This could be compared with 97 percent of those suffering from neck/back pain reporting production loss due to the problem. The average level of health-related production loss was 5.26 for the total population. The reported level of production loss among those with CMD was on average lower (4.62) than for people suffering from neck/back pain (6.34).

**Table 1:** Description of the study population

	Total population	CMD	Back/neck pain	Differences between groups
	*N* = 965	*N* = 556	*N* = 409	
Diagnostic groups, n (%)				
Back/neck pain	409 (42)	-	-	
CMD	556 (58)	-	-	
Sex, n (%)				*p* = 0.001^a^
Women	752 (78)	413 (74)	339 (93)	
Men	213 (22)	143 (26)	70 (17)	
Education, n (%)				*p* = 0.017^a^
University	298 (31)	191 (34)	107 (26)	
High school	539 (57)	302 (55)	237 (59)	
Preschool	119 (12)	60 (11)	59 (15)	
Age, mean (sd)	41.02 (11.5)	38.52 (11.8)	44.40 (10.2)	*p* < 0.001^b^
HRQL, m (sd), Range 0-1	0.55 (0.28)	0.63 (0.25)	0.45 (0.34)	*p* < 0.001^b^
General health, n (%)				*p* < 0.001^a^
Excellent	4 (0)	2 (0)	2 (0)	
Very good	64 (7)	54 (10)	10 (3)	
Good	215 (22)	165 (30)	50 (12)	
Somewhat good	450 (47)	246 (44)	204 (50)	
Poor	229 (24)	86 (16)	143 (35)	
Work ability, mean (sd), Range 0-10	4.49 (2.7)	5.19 (2.7)	3.54 (2.5)	*p* < 0.001^b^
Worked the previous 6 months, n (%)				*p* < 0.001^a^
Yes	692 (72)	434 (79)	258 (63)	
No	267 (28)	118 (21)	149 (37)	
Production loss, n (%)				*p* < 0.001^a^
Yes	619 (92)	372 (88)	247 (97)	
No	57 (8)	50 (12)	7 (3)	
Production loss, mean (sd), Range 0-10	5.26 (3.05)	4.62 (3.10)	6.34 (2.65)	*p* < 0.001^b^

*CMD* Common mental disorders

^a^ = Tested with Pearson Chi-square test

^b^ = Tested with Students *t*-test

### Convergent validity

The analysis of convergent validity was performed for both the baseline and the follow-up measurement (Table 2). The general health score, i.e., from SF-12, showed a moderate (baseline) to strong (follow-up) negative correlation with health-related production loss. HRQL (EQ-5D) had a strong negative correlation with health-related production loss in both the baseline and follow-up measurement. Work ability also showed a strong negative correlation with health-related production loss. Some differences were found between the two subgroups (Table 3). These results support a majority of *hypotheses H1a-H3c*, while some hypotheses were only partly supported (H1a, H1c and H2a).

**Table 2:** Convergent validity. Expected and observed correlations of health-related production loss with health indicators. Total population

Health-related production loss, Baseline	Expected correlation	Observed correlation	*P*-value
General health	Moderate (−0.30 – -0.50)	Moderate (−0.422)^a^	<0.001
HRQL	Moderate (−0.30 – -0.50)	Strong (−0.525)^b^	<0.001
Work ability	At least moderate (−0.30 – -0.50)	Strong (−0.691)^a^	<0.001
Health-related production loss, Follow-up			
General health	Moderate (−0.30 – -0.50)	Strong (−0.551)^b^	<0.001
HRQL	Moderate (−0.30 – -0.50)	Strong (−0.543)^b^	<0.001
Work ability	At least moderate (−0.30 – -0.50)	Strong (−0.650)^a^	<0.001

^a^Expectations confirmed

^b^Correlations stronger than expected

**Table 3:** Convergent validity. Expected and observed correlations of health-related production loss with health indicators. Different subgroups

Health-related production loss, Baseline, B/N Pain	Expected correlation	Observed correlation	*P*-value
General health	Moderate (−0.30 – -0.50)	Moderate (−0.323)^a^	<0.001
HRQL	Moderate (−0.30 – -0.50)	Moderate (−0.433)^a^	<0.001
Work ability	At least moderate (−0.30 – -0.50)	Strong (−0.638)^a^	<0.001
Health-related production loss, Follow-up, B/N Pain			
General health	Moderate (−0.30 – -0.50)	Moderate (−0.404)^a^	<0.001
HRQL	Moderate (−0.30 – -0.50)	Moderate (−0.430)^a^	<0.001
Work ability	At least moderate (−0.30 – -0.50)	Strong (−0.581)^a^	<0.001
Health-related production loss, Baseline, CMD			
General health	Moderate (−0.30 – -0.50)	Moderate (−0.408)^a^	<0.001
HRQL	Moderate (−0.30 – -0.50)	Moderate (−0.491)^a^	<0.001
Work ability	At least moderate (−0.30 – -0.50)	Strong (−0.690)^a^	<0.001
Health-related production loss, Follow-up, CMD			
General health	Moderate (−0.30 – -0.50)	Strong (−0.558)^b^	<0.001
HRQL	Moderate (−0.30 – -0.50)	Moderate (−0.484)^a^	<0.001
Work ability	At least moderate (−0.30 – -0.50)	Strong (−0.639)^a^	<0.001

^a^Expectations confirmed

^b^Correlations stronger than expected

### Responsiveness

Changes in health-related production loss were significantly associated with changes in general health, HRQL, and work ability (Table 4). This finding supports *hypotheses H4a-H4c*, indicating that the measure has external responsiveness. No differences were found for the two subgroups, indicating that the measure is responsive when used for the different types of health problems in this study.

**Table 4:** Responsiveness validity. Mean change in different health indicators, and results from regression analysis evaluating responsiveness

	Difference Follow-up-baseline	Total population	
	Mean (SD)	β	CI
*Health-related production loss*	−1.408 (3.406)		
General health	−0.456 (0.907)	1.463	1.130-1.797*
Sex		−0.178	−0.931-0.574
Age		−0.013	−0.040-0.014
Education		−0.097	−0.562-0.367
Diagnosis		−0.161	−0.789-0.466
HRQL	0.1458 (0.255)	−4.554	−5.769- -3.339*
Sex		−0.191	−0.967-0.585
Age		−0.007	−0.034-0.021
Education		0.096	−0.385-0.577
Diagnosis		−0.398	−1.046-0.251
Work ability	1.495 (2.55)	−0.715	−0.826- -0.605*
Sex		−0.241	−0.937-0.454
Age		−0.004	−0.029-0.021
Education		0.001	−0.426-0.429
Diagnosis		0.060	−0.522-0.642

*Significant at *p*-value ≤ 0.05

## Discussion

This study expands the existing research on a measure of health-related production loss developed in Sweden by examining its construct validity and responsiveness in a population suffering from persistent back/neck pain or CMDs. Based on previous research into the construct validity of health-related production loss evaluated in relation to health conditions, the investigated measure provided the expected results and confirmed a majority of the hypotheses. In some cases (4 out of 18), the observed correlations were stronger than expected. That 14 out of 18 hypotheses were confirmed indicates that the instrument has good construct validity. This study also showed that changes in health conditions and work ability were significantly related to changes in health-related production loss, which indicates that the measure also has good external responsiveness.

It is a challenge to evaluate presenteeism, and the resulting production loss, over time. Health could be a good indicator of presenteeism and production loss, but presenteeism could also be affected by other things such as feelings of insecurity, which could affect the strengths of association of health and presenteeism/production loss. Job insecurity, for example, is important for people in their decisions to go to work despite poor health [30]. If employees feel more insecure about their job, or if there were changes to the regulations governing the right to sick leave, this could affect the distribution of the ratings in both instruments. A wider distribution of the rating could result in stronger correlations. Therefore, an increase in correlation strength between general health and production loss from baseline to follow-up measurement does not necessarily mean that the instrument has poor construct validity. It could also be an indicator of some impact from external changes. A difference in strengths in correlation over time was also found in the validation study of WPAI [16], of which this item is based.

The correlation matrix of the measures indicates that when the health of the respondents improved, health-related production loss decreased. This pattern was the same for both groups of patients (CMD and back/neck pain). Even though the strength of the correlations was similar for the two subgroups, i.e., fulfilled the requirements for being moderately or strongly correlated, the correlation estimate was higher for the population with CMD than for the population with back/neck pain. From this point of view, the measure seems to capture health-related production loss better among individuals with CMD than among those with neck/back pain.

Work ability has rarely been evaluated in relation to health-related production loss. In one study it was shown that perceived work ability may be a robust indicator for assessing perceived production loss [22]. That study used estimates of work ability from a different population and modeled it against information on health-related production loss. The present study adds to current knowledge by also showing, using data collected in the same population, that work ability and health-related production loss are related to each other. Another study [23] validated a measure of work functioning, which is related to work ability and production loss, by testing its correlation with both WAI and production loss. Both WAI and production loss had a correlation with work functioning that was lower than that observed between WAI and production loss in the present study. Since no other study was found that has tested for the association between WAI and production loss, it is difficult to determine whether the correlations found in the present study were too strong to conclude good construct validity or if they were as strong as could have been expected, which would suggest good construct validity.

Work ability is a concept commonly used in Sweden to determine whether a person will be eligible for sick pay or not. The Swedish social insurance agency has begun specifically to evaluate whether people have the ability to work rather than evaluating their health status when deciding whether to grant an application for sick leave [31]. In the present study, work ability showed a stronger correlation with production loss than did other measures of health. Thus, if the measure of health-related production loss works as intended, work ability and health-related production loss capture the related dimensions and both measures could be used to assess people’s ability to perform at work. This also indicates that work ability is a better measure than health for capturing reductions in people’s ability to perform at work.

It is well known that the health of an individual is directly associated to performance [2, 32, 33]. People with health problems tend to be less productive than healthy employees. There also seems to be a difference in how different health conditions affect the ability to perform at work, at least in terms of the subjective performance level. In the present study, the average health-related production loss differed between the group reporting CMD and those experiencing back/neck pains. The average reduction in performance level was greater among people with back/neck pains. From both the company and societal perspective, this could indicate that, on an individual level, back/neck pain is a more costly health problem than CMD, as the individual’s ability to perform at work is affected to a greater extent. The average reduction in performance among individuals with back/neck pain in this population, who were recruited to obtain early interventions for their health problem to avoid sickness absence, or in some cases to return to work, was 6.34 on a scale from 0 to 10, where 10 means that health problems completely prevented them from working. The corresponding value for the individuals with CMD was 4.62. This scale is usually converted from 0 to 10 up to 0 to 100 in order to capture production loss in terms of a percentage [9]. This means that the difference between the two groups is more than 17 percent.

The measure used in the present study asks people to rate how health-related problems affected their ability to perform while at work over the previous seven days. This measure is derived from WPAI presenteeism dimension [16], but differs in asking whether health affects their performance rather than about the effect on productivity. WPAI is an instrument that has been used in several studies and is one of the most extensively tested work productivity instruments to date [34]. It has several strengths besides the rigorous validation testing, such as the inclusion of production loss measures in several domains and a short completion time. The limitations, however, are that is does not consider compensation mechanisms or work team dynamics [34] and that it only captures health-related production loss [11]. The latter is especially important if the purpose is to assess total production loss at the workplace, i.e., reduced performance due to work environment-related problems as well as health-related problems and absenteeism. This validation of the health-related production loss item is a first step towards creating a valid and reliable questionnaire consisting of those three dimensions (absenteeism, work environment-related loss and health-related loss) in order to capture a more comprehensive picture of production loss at the workplace. Previous validation studies on production loss instruments have mainly been conducted on cross-sectional data, while longitudinal validity tests barely exist [34]. In the present study validation is conducted using both cross-sectional and longitudinal data, indicating good validity also when considering responsiveness. The reliability of asking people to rate their own performance has been under discussion, with some questioning the accuracy of the information in relation to actual productivity. There have been suggestions that people tend to overrate their performance because they are afraid of telling the truth in case this information should become known to the employer, or that people overestimate their production loss and that the effect on output is lower than estimated [35]. In a previous study evaluating the construct validity of the Health Performance Questionnaire [36], the measure where people were asked to rate their own performance level was shown to have the highest correlation with both mental and physical health. This was compared to others measures where the employees were asked, for example, to rate their performance compared to other workers in similar occupations. These measures showed weaker correlations with both mental and physical health. The authors therefore suggest that this measure, where the employees rate their own performance alone, is sufficient when calculating the cost of presenteeism [36]. As the measure used in the present study is similar to the one in the Scuffham et al. study [36]—i.e., the individuals were asked to rate their own performance levels—, this could indicate that the measure is also suitable for calculating the cost of presenteeism. To be able to justify this conclusion, subjective measures of production loss need to be validated against objective measures of production.

### Limitations

As can been seen in Table 1, the individuals with CMD were more healthy than those with back/neck pain at baseline. They rated their general health as slightly better, reported better work ability on average and had fewer numbers reporting that they had not been working during the previous six months. Also, a larger proportion of them had a university education than in back/neck pain group. It is possible that the difference in production loss ratings is due to the severity of the problems resulting in long term sick leave for back/neck pain patients to a higher extent than for CMD patients. However, this problem should have a limited effect as we only used those who had had a job during the previous six months. It is also possible that the difference in production loss could be explained by the type of job, as well as by the ability of respondents to adapt their job tasks to their current state of health, which would result in lower levels of production loss, or that their ability to perform at work is not affected to the same extent as it is for back/neck pain patients. This could be indicated by the difference in educational level between the two groups. Unfortunately, no information about patients’ job types was collected in the study to enable testing for that. In a recently published study comparing production loss due to sick leave for patients with CMD and back/neck pain in a Swedish setting, differences were found between job types [37]. White-collar professions were more common in the group with CMD whereas blue-collar professions were more common in the back/neck pain group. This supports the explanation that the lower levels of production loss could be an effect of how people’s ability to perform is affected by the kind of job they have, and not only by their health.

Another limitation is that the data does not discriminate between those on full-time sick leave and those partly in work at baseline. The only information available is whether they have had a job during the previous six months. In this study the aim was to evaluate the validity of the instrument, i.e., if people’s ability to perform at work was correlated with their health. People who rate their production loss as high are assumed also to rate their health as poor, and a reduction in health-related production loss should be accompanied by an improvement in health and vice versa. A person on sick leave should theoretically quote a high loss or leave the question unanswered (since it specifically asks about the previous seven days), and at the same time rate their work ability or general health as poor. For this reason we don’t think this limitation affects the ability to use the data for validation of this measure, but we do think it is important to have this in mind when reflecting upon the results.

### Future research

This measure of production loss has so far been evaluated with regard to its construct validity and responsiveness. Future studies investigating its validity with regard to objective production data and to other validated performance measurements, as well as studies evaluating the stability of the measure, are needed.

## Conclusion

The present study demonstrates that this measure of health-related production loss is a valid measure capturing production loss due to illness, and that work ability is more strongly associated with health-related production loss than people’s general health is. Sickness presenteeism—that is, being sick but at work—is a costly matter for employers. The results of this study show an average of about 50 % reduced production due to illness among employees, with back pain being the most costly. The potential economic benefit of interventions to prevent work-related illness is highlighted by these results.

## Abbreviations

CMDs: Common mental disorders
HCU: Health care units
HRQL: Health-related quality of life
RW: Rehabilitation warranty
SF-12: Short Form-12
WAI: Work Ability Index
